# TRPV1 nociceptors are required to optimize antigen-specific primary antibody responses to novel antigens

**DOI:** 10.1186/s42234-024-00145-6

**Published:** 2024-05-29

**Authors:** Aisling Tynan, Téa Tsaava, Manojkumar Gunasekaran, Carlos E. Bravo Iñiguez, Michael Brines, Sangeeta S. Chavan, Kevin J. Tracey

**Affiliations:** 1 Institute for Bioelectronic Medicine, Feinstein Institutes for Medical Research, Northwell Health, Manhasset, NY USA; 2 The Elmezzi Graduate School of Molecular Medicine, Manhasset, NY USA; 3 Donald and Barbara Zucker School of Medicine at Hofstra/Northwell, Hofstra University, Hempstead, NY USA

**Keywords:** Nociceptors, Antibody, TRPV1, Neuroimmunology, Adaptive immune response, Optogenetic stimulation, Neuronalablation

## Abstract

**Background:**

Key to the advancement of the field of bioelectronic medicine is the identification of novel pathways of neural regulation of immune function. Sensory neurons (termed nociceptors) recognize harmful stimuli and initiate a protective response by eliciting pain and defensive behavior. Nociceptors also interact with immune cells to regulate host defense and inflammatory responses. However, it is still unclear whether nociceptors participate in regulating primary IgG antibody responses to novel antigens.

**Methods:**

To understand the role of transient receptor potential vanilloid 1 (TRPV1)-expressing neurons in IgG responses, we generated TRPV1-Cre/Rosa-ChannelRhodopsin2 mice for precise optogenetic activation of TRPV1 + neurons and TRPV1-Cre/Lox-diphtheria toxin A mice for targeted ablation of TRPV1-expressing neurons. Antigen-specific antibody responses were longitudinally monitored for 28 days.

**Results:**

Here we show that TRPV1 expressing neurons are required to develop an antigen-specific immune response. We demonstrate that selective optogenetic stimulation of TRPV1^+^ nociceptors during immunization significantly enhances primary IgG antibody responses to novel antigens. Further, mice rendered deficient in TRPV1- expressing nociceptors fail to develop primary IgG antibody responses to keyhole limpet hemocyanin or haptenated antigen.

**Conclusion:**

This functional and genetic evidence indicates a critical role for nociceptor TRPV1 in antigen-specific primary antibody responses to novel antigens. These results also support consideration of potential therapeutic manipulation of nociceptor pathways using bioelectronic devices to enhance immune responses to foreign antigens.

## Abstract (summary)

Genetic and selective functional evidence reveals that TRPV1^+^ nociceptors are required for primary IgG antibody responses to novel antigens and stimulating TRPV1^+^ nociceptors enhances primary IgG antibody responses to novel antigens.

## Introduction

Nociceptors are sensory neurons that respond to changes in the internal and external environment by eliciting defensive behavior and modulating immune responses. These specialized neurons densely innervate peripheral tissues, including the skin, joints, respiratory and gastrointestinal tract and are readily activated by various noxious stimuli such as chemicals, temperature, and pathogens (Baral et al. 2018a, b, 2019; Chiu et al. 2012). Nociceptors express molecular pattern receptors which enable the detection of pathogens and other inflammatory signals during infection and injury (Lai et al. 2017; Chiu et al. 2013; Hanes et al. 2016; Gunasekaran et al. 2018). Transient receptor potential vanilloid 1 (TRPV1) - expressing neurons are a subset of peripheral small- and medium-diameter nociceptors terminating in skin and soft tissues (Baral et al. 2019; Chiu et al. 2012). TRPV1^+^ nociceptors respond to a variety of stimuli including a number of exogenous (such as capsaicin, resiniferatoxin and some venom toxins) and endogenous (such as high temperature, acidic pH, ATP, lipoxygenase products, lipid metabolites, anandamide and monoacylglycerols) noxious stimuli (Szallasi and Blumberg 1999; Helyes et al. 2007).

Activation of the TRPV1 ion channel results in generation of an action potential that travels from the nerve terminal to the cell soma and the central nervous system (CNS) leading to pain sensation (Julius and Basbaum 2001; Scholz and Woolf 2007; Caterina and Julius 2001). In addition to transmitting pain signals to the CNS, activated TRPV1^+^ nociceptors send local efferent signals to release neurotransmitters, including substance P and calcitonin gene-related peptide (CGRP), into nearby tissues to modulate inflammatory responses (Szallasi and Blumberg 1999; Pinho-Ribeiro et al. 2018). By binding to cognate receptors on immune cells, these neuropeptides induce changes in cytokine production, immune cell transcriptome, and immune phenotype (Pinho-Ribeiro et al. 2017). Recent studies have also appreciated that TRPV1^+^ nociceptors are present in close proximity to dendritic cells (DC), and physically interact with DCs to modulate its function (Cohen et al. 2019; Kashem and Kaplan 2016; Kashem et al. 2015; Huang et al. 2021; Hanč et al. 2023). By releasing specific neuropeptides, nociceptors also regulate Th2 and Th17 type immune responses. In type 2 immune responses, TRPV1^+^ nociceptors act as primary sensors of allergens and release substance P, which stimulates migration of proximally located DCs to the local draining lymph nodes to initiate T helper-2 responses (Perner et al. 2020). On the other hand, TRPV1^+^ nociceptors can be directly activated by bacterial (Chiu et al. 2013) or fungal (Kashem et al. 2015) products to release CGRP which modulates neutrophil (Chiu et al. 2013) and CD301b^+^ dermal dendritic cell responses (Kashem et al. 2015).

Recent work shows that TRPV1^+^ nociceptors, in addition to modulating innate type 2 allergic responses, also affect the production of IgE (Mathur et al. 2021). Using models of airway and skin allergic inflammation, these studies showed that genetic or pharmacologic silencing of TRPV1^+^ nociceptors induces substantial reduction in IgE production (Mathur et al. 2021). IgE antibodies are well known for their role in mediating allergic reactions and differ from other antibody isotypes in being located predominantly in tissues at the sites of allergic reactions (Gould et al. 2003). Although these studies highlighted that selective silencing of TRPV1^+^ nociceptors attenuates the IgE responses, the role of TRPV1^+^ nociceptors on the development of circulating primary antigen-specific IgG responses to novel antigen was not previously clear.

Here we reasoned that TRPV1^+^ nociceptors play an important role in the development of primary IgG antibody responses to novel antigens. In this brief report using two different models of antigen immunization, we demonstrate that selective activation of TRPV1^+^ nociceptor neurons by optogenetic stimulation significantly enhances antigen-specific primary IgG antibody responses in the circulation. In addition, selective ablation of TRPV1^+^ nociceptors in mice exposed to a novel antigen significantly reduces primary IgG antibody responses.

## Results

### Optogenetic stimulation of TRPV1^+^ nociceptors enhances antigen-specific antibody responses

**Fig. 1:**
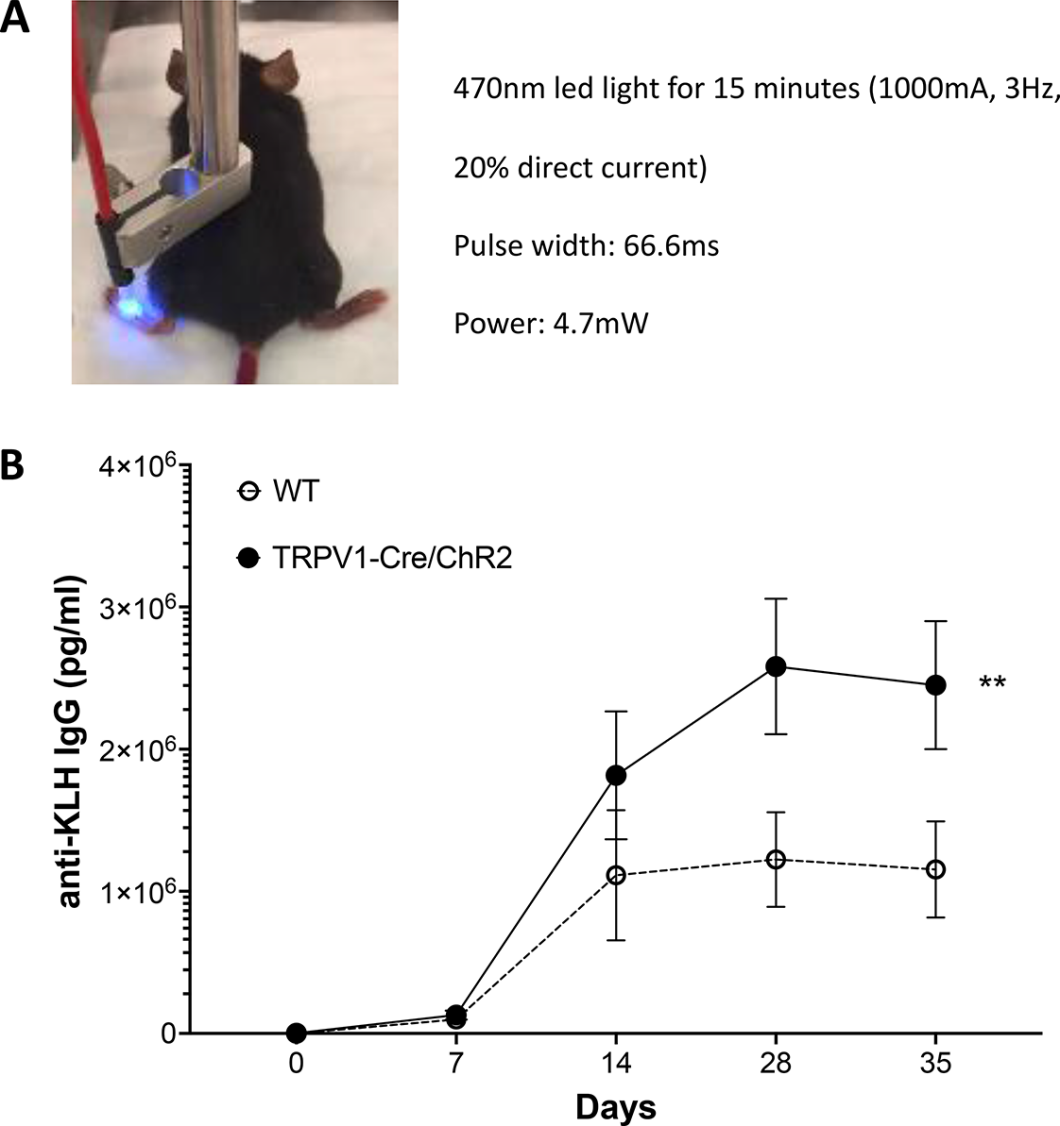
Selective optogenetic activation of TRPV1^+^ nociceptors enhances antigen-specific antibody response following immunization. TRPV1-Cre/ChR2 mice (*n* = 11) or C57BL/6J mice (*n* = 9) were subjected to optogenetic stimulation using blue light (473 nm, 3 Hz, 20% duty cycle, 15 min, 1000 mA) on the dorsum of the hind paw. **(A)** Representative image of the positioning of the 470 nm light source used to stimulate the dorsum of the hind paw. **(B)** Mice were immunized with 3 mg/kg KLH into the same paw immediately following optogenetic stimulation, and serum collected every 7 days. Serum was assayed for anti-KLH IgG antibodies by ELISA. Data are represented as mean ± SEM for each time point. Two-way ANOVA: wild-type versus TRPV1-Cre/ChR2 ; **p < 0.01

To establish a causal role for TRPV1^+^ nociceptors in enhancing antigen-specific antibody responses, we used an optogenetic approach. To specifically activate TRPV1^+^ nociceptors during immunization, we generated TRPV1-Cre/ChR2 mice by crossing TRPV1-Cre mice with Rosa26^ChR2-eYFP/+^ (ChR2) mice. Using blue light (450–490 nm, 3 Hz, 20% duty cycle, 4.7mW, 15 min), we optically stimulated dorsal paw innervating TRPV1^+^ nociceptors in TRPV1-Cre/ChR2 mice during antigenic challenge with keyhole limpet hemocyanin (KLH), a naturally occurring respiratory protein of giant keyhole limpets which is not ordinarily encountered by mammalian immune system (Harris and Markl 1999; Kantele et al. 2011), (Fig. 1A). As controls, C57BL/6J mice devoid of ChR2 expression were exposed to identical stimulation parameters. Optogenetic stimulation of TRPV1^+^ nociceptors significantly increases antigen-specific antibody responses in TRPV1-Cre/ChR2 mice as compared to controls (anti-KLH IgG ng/ml: TRPV1-Cre/ChR2: 2,581 ± 1,165, *n* = 6 versus Control: 1,224 ± 939, *n* = 8; *p < 0.05; Fig. 1B). These findings indicate that activated TRPV1^+^ nociceptors regulate the development of antigen-specific IgG antibody responses in mice.

### TRPV1^+^ nociceptors control onset of antigen-specific antibody responses

To confirm the role of TRPV1-expressing nociceptors in modulating antigen-specific primary IgG antibody responses, we utilized a genetic approach to selectively ablate TRPV1^+^ nociceptors by crossing TRPV1-Cre mice that expressed Cre recombinase under the control of the *TRPV1* locus with floxed diphtheria toxin A (DTA) mice (Chiu et al. 2013). TRPV1-driven expression of DTA induces cell death by catalyzing the inactivation of elongation factor 2 and inhibiting protein synthesis (Collier 2001; Maxwell et al. 1986; Palmiter et al. 1987), which results in the loss of TRPV1 + receptor neurons. These animals have a complete loss of thermal sensation, but otherwise appear phenotypically normal, including having intact touch and mechanical sensitivity (Mishra et al. 2011). We have previously confirmed that TRPV1-Cre/DTA mice lack TRPV1^+^ nociceptors (Zanos et al. 2018).

**Fig. 2:**
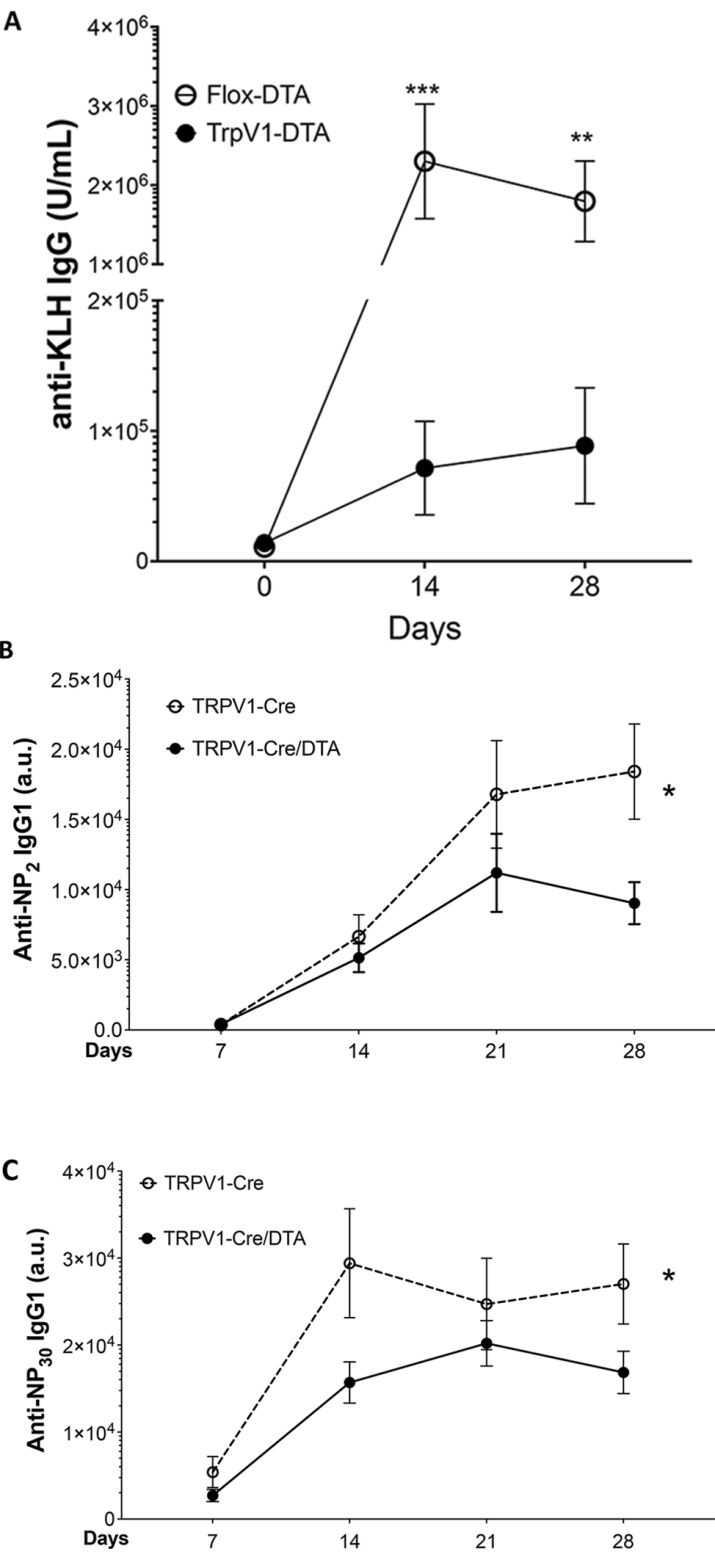
TRPV1-ablation impairs antigen-specific antibody response following immunization. **(A)** TRPV1-Cre/DTA (*n* = 7) or flox-DTA control mice (*n* = 5) were immunized with 4 mg/kg KLH intraperitoneally and serum was assayed for anti-KLH IgG antibodies by ELISA. Data are represented as individual mouse data points with mean ± SEM. Ordinary one-way ANOVA with multiple comparisons test between groups: flox-DTA control versus TRPV1-Cre/DTA; **p < 0.01, ***p < 0.001. **(B-C)** TRPV1-Cre/DTA mice or flox-DTA control mice were also immunized with NP-OVA in the foot pad and serum collected every 7 days. **(B)** High affinity and **(C)** low-affinity NP-specific IgG1 antibodies were quantified by ELISA. Data is represented as mean ± SEM for each time point. (*n* = 18/group from 2 repeated experiments). Two-way ANOVA: flox-DTA control versus TRPV1-Cre/DTA; *p < 0.05

We then immunized flox-DTA control and TRPV1-ablated (TRPV1-Cre/DTA) mice with KLH and monitored KLH-specific antibody responses in serum on day 0, 14 and 28 post-immunization. Ablation of TRPV1^+^ nociceptors in TRPV1-Cre/DTA mice results in significant reduction of KLH-specific IgG levels as compared to flox-DTA control mice for 28 days (anti-KLH IgG U/mL: D14: flox-DTA control, 2,300,803 ± 724,085, *n* = 4 versus TRPV1-Cre/DTA, 71,481 ± 35,903, *n* = 7, ***p < 0.001 and anti-KLH IgG U/mL, D28: flox-DTA control, 1,796,135 ± 509,461, *n* = 4 versus TRPV1-Cre/DTA, 88,633 ± 44,366, *n* = 7, **p < 0.01; Fig. 2A). Next, we assessed antibody responses to locally administered (4-hydroxy-3-nitrophenyl) acetyl (NP) hapten coupled to ovalbumin (OVA) carrier protein. Naive TRPV1-Cre control and TRPV1-ablated mice were immunized in the hind paw with 12.5 µg of NP-OVA, and blood samples were collected at 7,14, 21 and 28 days post-immunization. NP-specific IgG antibody responses were measured by ELISA using NP_2_-BSA and NP_30_-BSA to assess both high-affinity and low-affinity antibodies respectively (Reth et al. 1978). The titers of NP-specific high-affinity (anti-NP_2_) and low-affinity (anti-NP_30_) IgG antibodies are significantly reduced in TRPV1-ablated mice (Fig. 2B-C).

### TRPV1 does not alter T and B lymphocyte abundance or B cell class switching

**Fig. 3:**
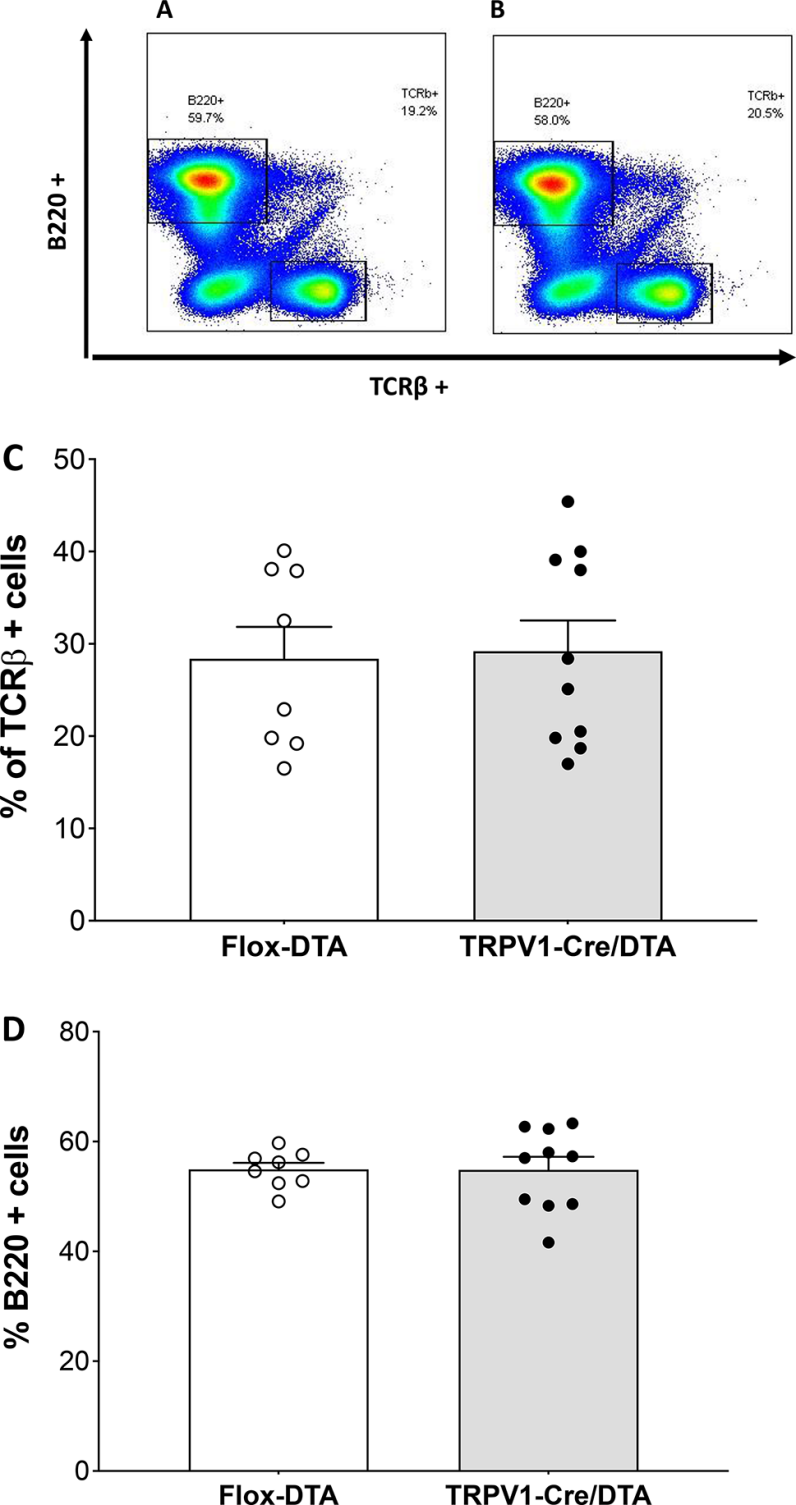
TRPV1 ablation does not change the frequency of T or B Lymphocytes in comparison to flox-DTA control mice. T cell and B cells were quantified in spleens isolated from flox-DTA control (*n* = 8) and TRPV1-Cre/DTA (*n* = 10) mice by flow cytometry. Representative scatterplots showing B220 + cells versus TCRβ + cells from **(A)** flox-DTA control and **(B)** TRPV1-Cre/DTA mice. The frequency of **(C)** TCRβ + cells or **(D)** B220 + cells revealed no difference between flox-DTA control and TRPV1-Cre/DTA mice. Data are represented as individual mouse data point with mean ± SEM. (*n* = 8–10/group from 2 repeated experiments)

TRPV1 expression has been suggested in immune cells such in T cells, macrophages, dendritic cells and mast cells (Helyes et al. 2007; Collier 2001), so it is possible that TRPV1-driven DTA expression in TRPV1-Cre/DTA mice may reduce T and B cell numbers. To address this possibility, TRCβ + T cell and B220^+^ B cell frequencies were analyzed in the spleen of flox-DTA control and TRPV1-ablated mice. No significant differences were observed in the frequency of B cells (flox-DTA control, 54.91% ± 3.389%, *n* = 8 versus TRPV1-Cre/DTA, 54.89% ± 7.42%, *n* = 10) or T cells (flox-DTA control, 28.38% ± 9.772%, *n* = 8 versus TRPV1-Cre/DTA, 29.2% ± 10.52%, *n* = 10) in TRPV1-ablated mice (Fig. 3A–D).

**Fig. 4:**
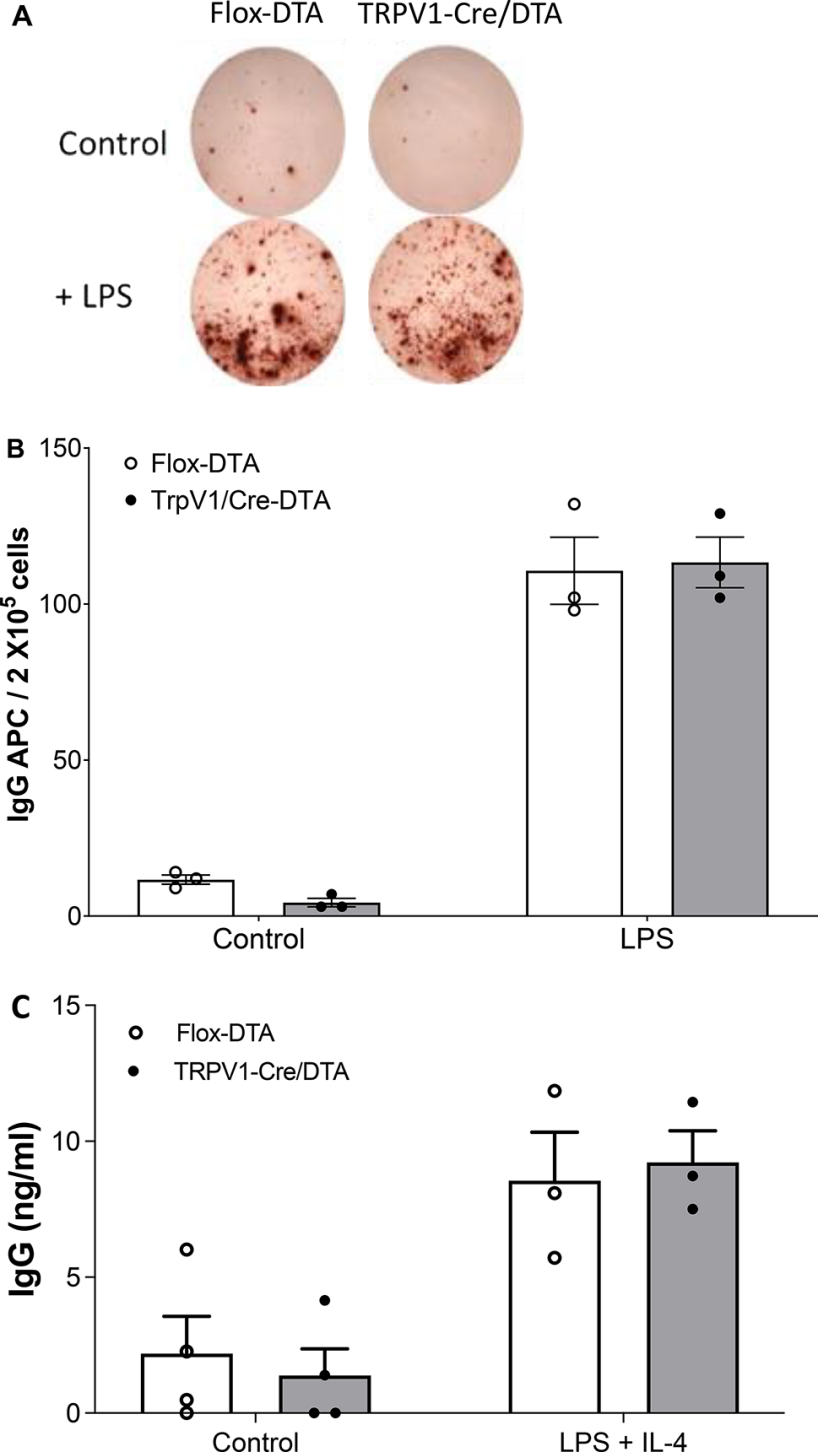
TRPV1 ablation does not impair the antibody response of in vitro activated splenocytes. Splenocytes from flox-DTA control or TRPV1-Cre/DTA mice were stimulated in vitro with LPS, and the number of antibody secreting cells (ASC) was determined by ELISpot assay on anti-IgG coated plates. (**A**) Representative images from total IgG ELISpot assay performed on splenocytes from flox-DTA control mice or TRPV1-Cre/DTA mice. Data are representative of 3 independent experiments. **(B)** Quantification of ASCs in unstimulated and LPS-stimulated splenocytes from flox-DTA control and TRPV1-Cre/DTA mice using anti-IgG ELISpot assay. **(C)** Supernatant from unstimulated or LPS-stimulated splenocytes from flox-DTA control and TRPV1-Cre/DTA mice was collected after 72 h. Levels of total IgG secreted was measured using ELISA. Data are represented as individual data point for each experiment with mean ± SEM. Each experiment was carried out with at least 3 replicates per experiment

To determine whether B cells harvested from TRPV1-ablated mice are capable of producing an antibody response, we counted number of antibody secreting cells (ASC) in total splenocytes from flox-DTA control and TRPV1-ablated mice. No significant difference in the number of antibody secreting cells is observed in unstimulated or LPS-stimulated splenocytes from TRPV1-ablated mice as compared to flox-DTA control mice (Fig. 4A-B; splenocytes + LPS: IgG^+^ASC/2 × 10^5^ cells, flox-DTA control mice: 111 ± 19 versus TRPV1-Cre/DTA: 113 ± 14, *n* = 3 experiments each with *n* = 3 replicates each). Comparable levels of secreted IgG are observed in the cell supernatants (Fig. 4C; IgG ng/ml: flox-DTA control, 7.8 ± 4.7 versus TRPV1-Cre/DTA, 7.8 ± 3.6, *n* = 4 experiments each with *n* = 3 replicates each).

## Discussion

Nociceptor neurons interact with innate immune cells to regulate immune responses and protect organisms from dangerous stimuli. It is potentially advantageous that interactions between nociceptors and immune cells shape host responses to defend from pathogens. Although, TRPV1^+^ nociceptors play a critical role in mediating innate immune cell responses (Chiu et al. 2013; Pinho-Ribeiro et al. 2018) whether they also modulate antigen-specific primary antibody responses was previously unknown. Here, we utilized an in vivo optogenetic strategy to target TRPV1 + nociceptors and show that selective TRPV1^+^ neuron activation enhances antigen-specific primary IgG antibody responses. Optogenetic stimulation of cutaneous TRPV1^+^ nociceptors was sufficient to induce a time-dependent increase in KLH-specific IgG responses. Moreover, we found that selective genetic ablation of TRPV1^+^ nociceptors abrogated the increase in antigen-specific IgG responses.

Although absence of TRPV1^+^ nociceptors accomplished via genetic ablation or pharmacological silencing was previously shown to reduce number of IgE-secreting B cells and local IgE levels in allergic inflammation (Mathur et al. 2021), it was previously unknown whether nociceptor signals are required for the development of antigen-specific primary IgG antibody responses. To put the current studies in perspective, TRPV1^+^ nociceptors potentiate adaptive immune responses in atopic dermatitis and psoriasis (Riol-Blanco et al. 2014; Wilson et al. 2013). Cutaneous TRPV1^+^ neuron activation is sufficient to trigger inflammation and augment local host defense (Cohen et al. 2019). Genetic ablation or pharmacological disruption of TRPV1 attenuates airway injury and asthma in murine models of allergic airway inflammation (Rehman et al. 2013; Mabalirajan et al. 2013) while TRPV1 denervation decreases dendritic cell numbers in response to antigenic challenge (Kradin et al. 1997). Administration of capsaicin in cytotoxic doses to rat neonates reduces antigen-specific antibody secreting cells as observed by direct and indirect plaque assay methods (Helme et al. 1987a, b), whereas capsaicin administration reduces IgA and IgG synthesis in cultured lymphoid cells (Nilsson et al. 1991).

Although immune cells have been reported to express TRPV1 (Bertin et al. 2014; Omari et al. 2017), other independent studies analyzing transcriptome data sets in the Immunological Genome Project and a gene atlas of protein-encoding transcriptomes showed that TRPV1 expression is absent in immune cells (Baral et al. 2018a, b). Careful analysis of expression of TRPV1 using reporter mice further confirmed the selective expression of TRPV1 in neuronal cells and not in non-neuronal cell types (Cohen et al. 2019). Additionally, prior work has shown that unchallenged TRPV1-Cre/DTA mice have normal appearing germinal centers in the spleen and pulmonary lymph nodes (Mathur et al. 2021). Consistent with these studies, we did not find any significant decrease in total B and T lymphocyte numbers in TRPV1-Cre/DTA mice. In addition, no significant difference was observed in the number of germinal center B cells in both naïve and immunized TRPV1-DTA mice at day 21 post-vaccination. B lymphocytes from TRPV1-Cre/DTA mice are functionally competent given that splenocytes isolated from naïve TRPV1-Cre/DTA mice readily class-switch and produce IgG in vitro when stimulated with typical class-switch stimuli (LPS + IL-4). Although we cannot exclude the possibility that TRPV1^+^ lymphocytes are somehow impaired in TRPV1-Cre/DTA animals, the available evidence indicates that: (1) optogenetic stimulation of TRPV1^+^ nociceptors enhances antibody responses; (2) T and B cells counts are not significantly changed in TRPV1-Cre/DTA animals as compared to controls; and (3) B cells from the TRPV1-Cre/DTA animals maintain their capacity to undergo class switching. Together, this suggests a major mechanistic role for TRPV1^+^ nociceptors in mediating antigen-specific antibody responses.

There are several important implications from these results. First, this reveals a nervous and immune system mechanism responsible for primary antibody production during encounters with novel antigens. Understanding immune responses to novel antigens requires more than understanding solely mechanisms of dendritic cells, macrophages and lymphocytes. It also requires understanding these nociceptor mechanisms. It will be interesting to consider further how nociceptors contribute to responses to novel antigens. Second, understanding that specific stimulation of nociceptors by optogenetic technology enhances antibody responses offers a novel insight into targeting nociceptors during immunization. As previously noted by Charles Janeway, antigen by itself is not sufficient to activate an adaptive immune response: signals from host cells, such as elicited by adjuvants, are required (Janeway 1989). Because adjuvant administration mediates painful inflammation and necrosis, it is now important to revisit the role of nociceptor signaling in adjuvant mechanisms of immunity.

There are some limitations of this study. First, the mechanism of the beneficial effects of activation of TRPV1^+^ nociceptors on IgG antibody production is currently unresolved. In these experiments, changes in IgG levels was only studied in response to activation or ablation of nociceptor TRPV1^+^. Second, in a primary immune response, IgM antibodies appear between days 4–7, several days before the detection of IgG. These studies focused on changes in circulating levels of IgG, and not on the GC B cells or IgM / IgG secreting plasma cells. Therefore, investigation of more detailed kinetics of the response as well as potential changes in other immunoglobulin classes will be illuminating. Third, the effect of TRPV1^+^ nociceptor activity on the maturation of B cells and class switching from IgM to IgG is currently unknown, indicating another area for further study. Fourth, the biological implications of augmented IgG responses in terms of resistance to infection should be evaluated in follow-on investigation. However, irrespective of these caveats the demonstration of a specific neuroimmune circuit which mediates IgG response to antigen presentation opens the possibility for direct modulation this response employing the technology of bioelectronic medicine.

## Materials and methods

### Animals

All procedures with experimental animals were approved by the Institutional Animal Care and Use Committee and Institutional Biosafety Committee of the Feinstein Institute for Medical Research, Northwell Health, Manhasset, NY in accordance with the National Institutes of Health Guidelines. Animals were maintained at 25 °C on a 12-hour light-dark cycle with free access to food and water. C57BL6/J, *R26*^LSL-DTA^ (B6.Cg-*Gt(ROSA)26Sor*^tm2.1(CAG-EGFP,-DTA*G128D)Pjen^/J), *R26*^LSL-ChR2^ (B6.Cg-*Gt(ROSA)26Sor*^tm32(CAG-COP4*H134R/EYFP)Hze^/J) and TRPV1-Cre (B6.129-*Trpv1*^*tm1(cre)Bbm*^/J) mice were purchased from Jackson Laboratory (Jackson Laboratory, Bar Harbor, ME, USA) and maintained in fully accredited facility at the Feinstein Institutes for Medical Research. TRPV1-Cre mice were crossed to *R26*^LSL-DTA^ mice or to *R26*^LSL-ChR2^ to generate TRPV1-Cre/DTA or TRPV1-Cre/ChR2 mice respectively. 6-12-week-old age-matched female and male mice were used for experiments.

### Optogenetics

To activate TRPV1^+^ nociceptors by blue light (473 nm; power 4.7 mW; frequency 3 Hz; 20% duty cycle, pulse width 67ms), a LED driver (Thor Labs, Newton, New Jersey) was connected to a blue LED source (Thor Labs). Parameters previously shown to demonstrate hypersensitivity in the paw for up to 24 h were used (Daou et al. 2013). The power from the LED was measured using a power meter with the LED positioned at the same 1 cm distance from the sensor at the start and end of the experiment. The power measured from these settings at both points was 4.7mW and the pulse width was 67ms. The LED was positioned over the right hind paw at 1 cm distance from the skin (Fig. 1A). TRPV1-Cre/ChR2 mice or C57Bl6 mice were stimulated with blue light on the hind paw for 15 min. Immediately following optogenetic stimulation, mice were injected with 3 mg/kg of KLH subcutaneously in 20 µL into the same paw. Blood was collected weekly, and serum antibody titers tested.

### Immunizations

For systemic immunization, mice were immunized with 4 mg/kg of Keyhole limpet hemocyanin (KLH) (Millipore Sigma, MA, USA) in 200 µl of saline by intraperitoneal injection. For local immunizations, mice were immunized with either 12.5 µg of NP_25_-OVA (LGC Bioresearch Technologies, USA) in 25ul PBS with Imject™ Alum (ThermoFisher Scientific, MA, USA) (2:1 ratio) in both hind paws or with 3 mg/kg KLH on one hind paw that was exposed to optogenetic stimulation. Blood samples were collected from immunized mice every 7 days, and levels of antigen-specific antibodies were determined by ELISA.

### Enzyme-linked immunosorbent assay (ELISA)

Anti-KLH IgG were measured using commercially available ELISA as per the manufacturer’s protocol (Abnova, Taiwan). Total IgG was assayed using Invitrogen murine Total IgG kit (ThermoFisher Scientific). High-affinity and low-affinity NP-specific antibodies were measured by ELISA using 10 µg/ml of NP_2_-BSA or NP_30_-BSA respectively as the coating reagent as previously described (Reth et al. 1978, Ersching et al. 2017). Briefly, serum was assayed in 4-fold dilutions starting at 1/100. NP-specific IgG1 was detected using a biotin conjugated rat anti–mouse IgG1 antibody (BD Biosciences, CA, USA) and developed with Strep-horseradish peroxidase (R&D Systems, MN, USA) and tetramethylbenzidine (Sigma, MO, USA). OD450 was measured using a Tecan sunrise absorbance microplate reader (Tecan, Switzerland). Titers were calculated by logarithmic interpolation of the dilutions with readings immediately above and immediately below an OD450 of 0.3.

### Cell preparation

Spleens were isolated from flox-DTA control or TRPV1-Cre/DTA mice and dissociated to a single cell suspension using a 70 μm cell strainer. Red blood cells were lysed and the remaining cells used for culture experiments or flow cytometry.

### Flow cytometry

Total splenocytes were incubated in PBS containing 2% fetal bovine serum and 2 mM EDTA (Gibco, ThermoFisher, USA). Fc receptors were first blocked using unconjugated anti-CD16/32 prior to staining with the following antibodies: B220-FITC (RA3-6B2), and TCRβ-BV510 (H57-597). All antibodies were from Biolegend (CA, USA). Dead cells were excluded using LIVE/DEAD Fixable Near-IR Dead Cell Stain kit (ThermoFisher). All analyses were performed using FlowJo software (Treestar, USA).

### Cell culture

Total splenocytes were cultured into 96 well tissue culture plates in triplicate at 2 × 10^5^ cells in RPMI 1640 (Gibco) supplemented with 1 M non-essential amino acids, 1 M Hepes, 1 M Glutamax, 50µM β-ME2, 1% Pen/Strep and 10% FBS (all Gibco). Cells were stimulated with 500 ng/ml LPS (*Escherichia coli* 0111:B4, Sigma) for 72 h. Supernatants were harvested for ELISA detection of IgG.

### Enzyme-linked immunosorbent spot (ELISpot)

Total splenocytes were cultured on anti-IgG ELISpot plates and developed using the standardized ELISpot kit (Mabtech Inc., Cincinnati, OH, USA). Briefly, 96-well flat bottom multi-screen filter plates (Millipore, Billerica, MA, USA) were coated with 100 µL per well of anti-mouse IgG antibody (15 µg/ml). The plates were incubated overnight at 4 °C, washed with PBS, and blocked using complete RPMI medium. Splenocytes were cultured at 2 × 10^5^ cells in triplicates in the plates overnight at 37 °C with 5% CO_2_. ELISpots were developed as per the manufacturer’s protocol, scanned, and analyzed at Cellular Technology Ltd. (Shaker Heights, OH, USA).

### Statistics

Data were analyzed using Graphpad Prism 10 software using two-tailed unpaired Student’s *t*-tests or 2way ANOVA with Sidak’s multiple comparisons test or 1way ANOVA with Dunn’s multiple comparisons test. For all analyses, *P* ≤ 0.05 was considered statistically significant.

## Data Availability

All data is included in the manuscript.

## Abbreviations

ANOVA: Analysis of Variance
ASC: Antibody Secreting Cell
BSA: Bovine Serum Albumin
ChR2: Channelrhodopsin-2
DC: Dendritic cell
DTA: Diphtheria toxin fragment A
EDTA: Ethylenediaminetetraacetic acid
ELISA: Enzyme-Linked Immunosorbent Assay
ELISpot: Enzyme-linked Immunosorbent Spot
FBS: Fetal Bovine Serum
Hz: Hertz
Ig: Immunoglobulin
KLH: Keyhole limpet hemocyanin
LED: Light-Emitting Diode
LPS: Lipopolysaccharide
NP: 4-Hydroxy-3-nitrophenylacetyl hapten
OVA: Ovalbumin
PBS: Phosphate Buffered Saline
TCRβ: T Cell Receptor Beta
TRPV1: Transient Receptor Potential Vanilloid 1
